# Automatic Hybrid Access Control in SCADA-Enabled IIoT Networks Using Machine Learning

**DOI:** 10.3390/s23083931

**Published:** 2023-04-12

**Authors:** Muhammad Usman, Muhammad Shahzad Sarfraz, Usman Habib, Muhammad Umar Aftab, Saleha Javed

**Affiliations:** 1 Department of Computer Science, National University of Computer and Emerging Sciences, Islamabad, Chiniot-Faisalabad Campus, Chiniot 35400, Pakistan; 2 AI and Data Science Department, FAST School of Computing, National University of Computer and Emerging Sciences, Islamabad, Islamabad Campus, Islamabad 44000, Pakistan; 3 Machine Learning Group, SRT, Lulea Technical University, 97187 Lulea, Sweden

**Keywords:** Industrial Internet of Things (IIoT), privacy preservation, resource-constrained IoT, access control, role propagation, industry 4.0, Internet of Things (IoT), deep learning

## Abstract

The recent advancements in the Internet of Things have made it converge towards critical infrastructure automation, opening a new paradigm referred to as the Industrial Internet of Things (IIoT). In the IIoT, different connected devices can send huge amounts of data to other devices back and forth for a better decision-making process. In such use cases, the role of supervisory control and data acquisition (SCADA) has been studied by many researchers in recent years for robust supervisory control management. Nevertheless, for better sustainability of these applications, reliable data exchange is crucial in this domain. To ensure the privacy and integrity of the data shared between the connected devices, access control can be used as the front-line security mechanism for these systems. However, the role engineering and assignment propagation in access control is still a tedious process as its manually performed by network administrators. In this study, we explored the potential of supervised machine learning to automate role engineering for fine-grained access control in Industrial Internet of Things (IIoT) settings. We propose a mapping framework to employ a fine-tuned multilayer feedforward artificial neural network (ANN) and extreme learning machine (ELM) for role engineering in the SCADA-enabled IIoT environment to ensure privacy and user access rights to resources. For the application of machine learning, a thorough comparison between these two algorithms is also presented in terms of their effectiveness and performance. Extensive experiments demonstrated the significant performance of the proposed scheme, which is promising for future research to automate the role assignment in the IIoT domain.

## 1. Introduction

The Internet of Things (IoT) is rapidly expanding, bringing forth a transformation in every aspect of our everyday lives. In the IoT paradigm, many objects in the environments are interconnected in the form of a network in one way or another [1]. The IoT’s development is a complex, large-scale process of technological innovation. At the outset of the IoT’s implementation, operating a domain-specific application was the primary development approach [2]. This application can be a production control system with industrial control and monitoring capabilities that provides multiple enterprise-related services. IoT applications are currently deployed in cross-industry applications based on the principles of public information services. In large-scale contexts, communication controllers and solution providers regulate and create these IoT applications, which support residential and industrial users. IoT-enabled applications are capable of location sensing [3], location information sharing [4], environment sensing [5], ad hoc networking [6], secure communication [7], remote operations and many more and are even capable of different service requirements [8].

The integration of the IoT for business automation is referred to as the Industrial Internet of Things (IIoT). However, when applied to critical infrastructures, the IIoT can expose them to severe network vulnerabilities, posing a disruptive threat to society [9]. SCADA systems are commonly utilized in such critical infrastructures to better supervise and control such IIoT application cases. Further, due to open standard protocols being used for communication between the core components, it is more vulnerable to security risks and threats. To deal with the security issues of SCADA-based networks, different types of security techniques are introduced and proposed in the literature including key management for the securing communication protocols [10,11,12], intrusion detection approaches [13,14], secure transmission of information [12], and access control strategies [15]. Among all the techniques, access control acts as the front-line security mechanism for the systems under threat. Access control has gained much importance in ensuring the prevention of information leakage by monitoring the access of data or resources and preventing the unauthorized transmission of information in SCADA [16]. The decision to allow access to a resource is known as access control [16].

In the existing literature, the roles are either assigned to users by network administrators manually or based on the attributes of the users. The assignment of roles to end objects by administrators is referred to as role engineering. The manual establishment of the roles by the network administrators is a tedious process with an impact on the efficiency of the overall process. Likewise, the situation becomes more complex in IIoT use cases due to the presence of heterogeneous devices interacting with each other and the hyperdynamic environment changing rapidly. The dynamic environment rapidly changes the characteristics of end objects in critical infrastructure applications such as metro transportation and industrial automation scenarios. In an attempt to resolve this issue, we propose a framework to automate role engineering using machine learning in access control. We propose two machine learning approaches to automatically execute the role engineering in a complex scenario with heterogeneous devices and changing environments. Following is the summary of the contributions of this proposed study:

1. We provide a detailed analysis of the current trends, gaps, and problems in access control approaches. We present a comparative study of all contemporary access control approaches with respect to the IIoT domain. The study offers a core understanding of the requirements of modern loosely coupled critical infrastructures.
2. We propose a framework for automatic role assignment problems in fine-grained access control. In existing studies, this process is manually adopted, which is a laborious and cumbersome process. By this, the fine-grained access control can achieve maximum flexibility in a time-efficient manner.
3. We leveraged supervised machine learning approaches to map the SCADA-based IIoT system for this problem, which is novel and open to further research. We employed machine learning to automatically execute the role assignment and propagation in such an environment that is changing and generating complex data.
4. We provide a thorough analysis of machine learning mapping to this domain with different hyperparameters and their effect to achieve maximum accuracy. A detailed discussion is provided in later sections of this paper.
5. A thorough comparison is presented between MLP and ELM based on validation, test, and time effectiveness. A different number of hyperparameters were considered in the environmental setup to conduct the experimental results.

The organization of the paper is as follows: First, we discuss the preliminary concepts of SCADA and access control in the following section. The state-of-the-art access control approaches with contemporary trends and open problems are discussed in detail in the literature review followed by the problem formulation for this research study. After that, we discuss the machine learning algorithms employed in this study in the proposed solution section followed by the environment setup section. After that, we discuss the results and performance measurements in the Results Section. Following that, the conclusion and future work are provided at the end.

## 2. Preliminaries

This section provides a preliminary understanding of the core components of the IoT environment and applications of access control.

### 2.1. Supervisory Control and Data Acquisition

**Figure 1:**
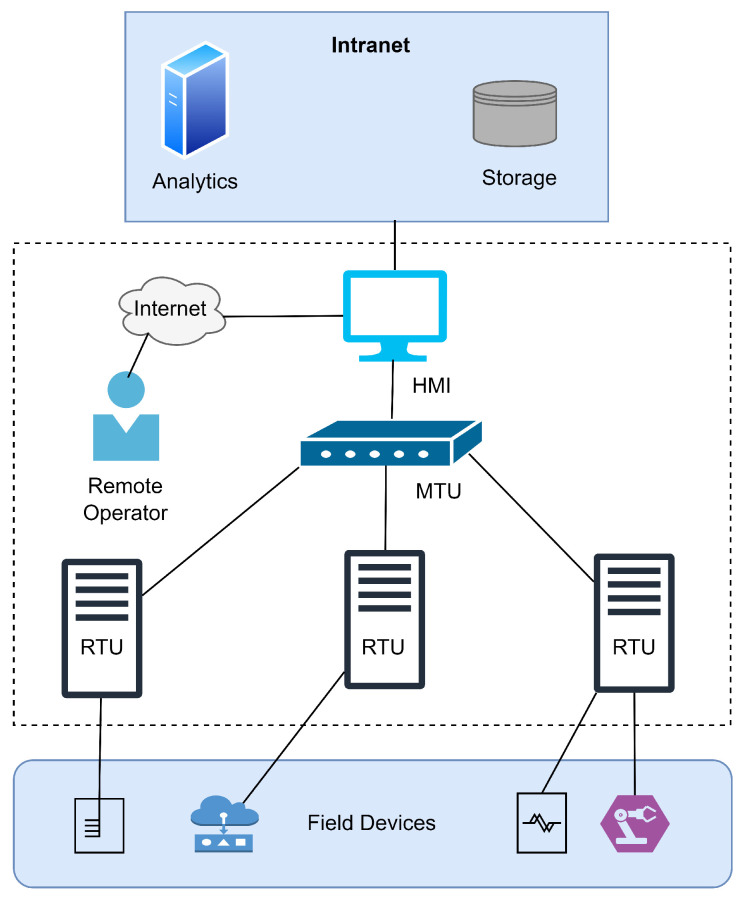
A naive architecture representation of SCADA network application with multiple control and monitoring services.

The basic operation of SCADA is to gather real-time information or data and monitor equipment and processes in the critical framework, providing the connection among servers located in the field or at remote locations [17]. Three major components involved in the architecture of the SCADA systems are intelligent electronic devices (IEDs), substation controllers, and power equipment. The transfer of commands or data may be carried between the substation controller and IED, between the IED and sensors, or between different IEDs transferred over the SCADA network. However, SCADA systems are now no longer considered isolated networks prohibiting outsiders from entering the network and also not private or specialized networks, allowing only authorized or related staff to access the resources [18]. Figure 1 shows the typical SCADA-based application architecture with its core components. The following section discusses the architecture and core components in detail.

#### 2.1.1. Architecture and Core Components

A traditional SCADA system consists of a central controller and a number of devices including sensors and actuators. They are widely used in industrial areas for controlling the process of the systems. It is composed of the following main components.

##### Operator

This is the one operating the system from the organization’s premises or remotely through the Internet. The major responsibilities of the operator are to monitor the system, alert addressing, and manage important control operations.

##### Human–Machine Interface

This is used to provide the interaction between the SCADA system and operator by collecting the information from the master terminal unit (MTU) and translating the commands to control properly.

##### Intranet

This is composed of computational networking and storage components that are located within the association.

##### Master Terminal Unit (MTU)

This transfers the information and control signals gathered from the remote terminals to the HMI, thus providing a high-level control logic in the system.

##### Remote Terminal Unit (RTU)

This provides the service of exchanging information and commands with the MTU and transferring specific control signals to field devices such as sensors and actuators.

##### Field Devices

These consist of devices that can monitor and control the process of the organization and distribute it in the organization. These devices include a number of sensors and actuators used for data collection and control actions’ execution, respectively.

### 2.2. Access Control

**Figure 2:**
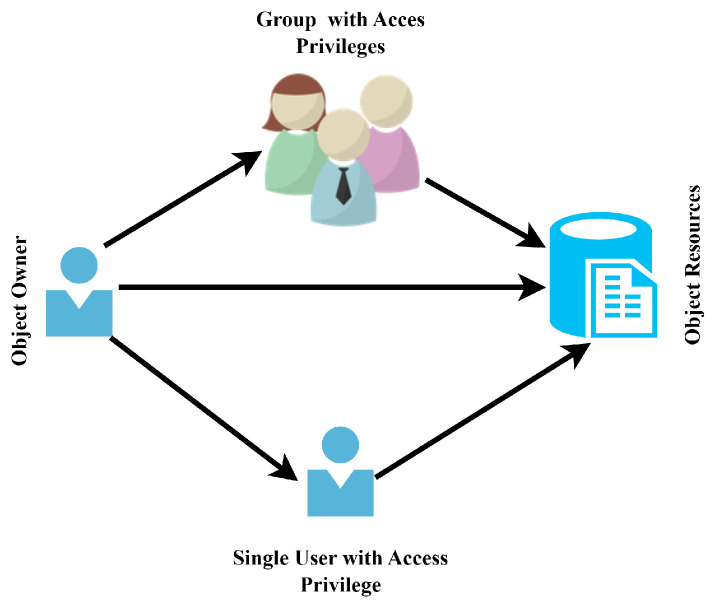
View of discretionary access control system architecture.

Access control is a process to control who can perform a particular task and which access rights a user can have on a specific resource. This access can be controlled by a subject, which can be a user, a device, or even a service. Different types of access control models have been proposed up till now, and much work is being performed in this area. According to Trusted Computer System Evaluation Criteria (TCSECs), there are two types of access control: discretionary access control (DAC) and mandatory access control (MAC) [19]. In DAC, the concept of ownership is used in which a user has ultimate control over his/her resources and he/she can permit access to other users over his/her resources or devices. Therefore, it is also considered an identity-based access control in which access rights are decided based on the identities of the users [20]. This model is able to meet the security needs; however, it also requires manually managing the users, authorities, and resources or devices, which causes difficulties in complex architectures. Figure 2 represents the naive representation of the DAC architecture. However, MAC is different from DAC as it is based on a set of rules by the system or a central authority, which is defined based on labels associated with requesting users and resources in naive terms [20].

**Figure 3:**
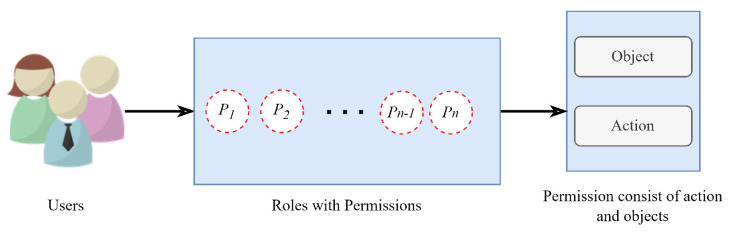
Naive representation of role-based access control system architecture.

**Figure 4:**
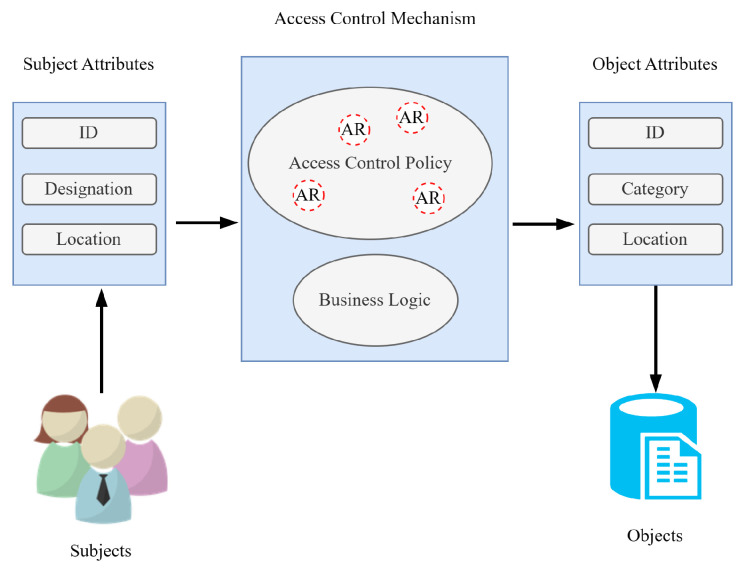
A naive architectural representation of attribute-based access control system consisting of groups of objects and subjects with attributes.

Although MAC is able to overcome the issues of DAC by centralizing the management, it is still not efficient enough to meet the performance requirements of complex environments [21]. In dynamic environments, there are many users participating in different tasks requiring an instant role shift. These role shifts can further require new object access rights for different users. To solve this problem, the role-based access control (RBAC) model was introduced, which relies on the restriction of resources accessible to authorized users. It is composed of three basic components, which are user–role, permission–role, and role-to-role relationship, helping to perform the user assignments in an easy way [21]. Figure 3 represents the naive architecture of the RBAC model. All of the access control models discussed so far are considered suitable for closed environments and are not able to adapt to modern loosely coupled computing environments. Therefore, to resolve this issue, the attribute-based access control (ABAC) model is developed, which relies on granting access to the requested resource based on the attribute assigned to the requesting user, resources, and environmental conditions and a set of rules related to those attributes and conditions. ABAC has been considered an appropriate model in the computing environment of today’s era having a vast range of applications [21]. Figure 4 represents the ABAC model. In the following section, the detailed working of the essential components of SCADA is provided.

## 3. Literature Review

The core concept of RBAC according to the NIST-RBAC-2000-standard [22] is that users and permissions are assigned to roles and users as members of roles obtain permissions. The relationship between user–role and permission–role in the RBAC model can be many-to-many. A novel access control model based on the RBAC framework was proposed in [23] using the semantic business roles and intelligent agents to implement intelligent RBAC (I-RBAC). A real dataset for occupational roles from Standard Occupational Classification (SOC) was used in this paper. This framework provides the required level of access control for a multi-domain environment with a highly dynamic nature by applying real-world semantic business roles and intelligent agent technologies. The authors in [24] proposed a platform using Ethereum’s smart contract technology to identify the role of the trans-organizational environment based on the RBAC model called RBAC-SC. Ethereum is a secure, flexible open blockchain platform in which smart contracts are established to provide decentralized applications serving as autonomous agents, which operate the same as programmed and installed on a blockchain. The authors of [25] considered the security issues of the Modbus, protocol which is used by most SCADA applications, and proposed a secure RBAC model to provide authorization to the client, as well as the Modbus frame. The Transport Layer Security (TLS) protocol was used to achieve authentication in the system after the completion of certificate verification at two endpoints.

In ABAC, authorization policies to determine an access decision are specified using the attributes or characteristics of objects in an access event. In order to mitigate the limitations of RBAC, the authors in [26] proposed a novel ABAC-based access control that is more flexible to serve the needs of IoT use cases such as smart devices and make the data exchange more secure in a cloud–IoT environment.

**Figure 5:**
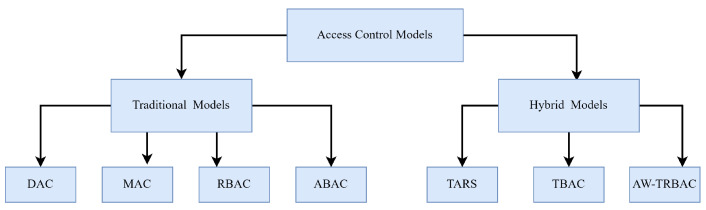
Classification of access control mechanisms currently being adopted in cross-domain applications.

Another ABAC-based model was presented in [27] for managing shared IoT devices in smart cities. In this model, the users hold their attributes and request authorizations by using diverse entities by setting up smart contracts. At the time of access, a trust level is calculated for each attribute whose value is dependent on the combined trust of each approving entity. The authors in [28] also proposed a formal ABAC model named ITS-ABACG to address the issues related to access control in the Industrial Internet of Vehicles (IIoV). The concept of groups was introduced in the proposed model, which is used to assign different smart entities according to different attributes such as location, direction, speed, and some others. A taxonomy of current access control methods that are being adopted in cross domain applications is presented in Figure 5.

Different types of approaches for privacy preservation have been proposed in the field of electronic health record (EHR) systems. For example, in [29], the authors proposed an ABAC model based on the Extensible Access Control Markup Language (XACML) for cloud-based EHR systems using XML encryption and XML digital signature techniques. A novel ABAC approach was proposed in [30] based on blockchain technology for IoT systems. This scheme has overcome the problem of maintaining an access control list for individuals in the system. According to the system, every device is defined by a set of predefined attributes, which are issued by the attribute authorities based on its identity or capability. For this purpose, the record of attribute distribution is stored using a blockchain.

However, to resolve the limitations of both ABAC and RBAC models, the authors in [31] proposed a hybrid approach for access control named hybrid access control (HAC), which is based on the dynamic conflict of interest (COI) on the level of the role to provide secure localization of vehicles based on the IoT and satellites. This hybrid model is the combination of the ABAC and RBAC models, and new attributes of RBAC entities are added, hence extending the RBAC model. A novel and dynamic access control model named authorizing workflow task role-based access control (AW-TRBAC) was proposed in [32], which is based on the dynamic segregation of duties (SoD) and process workflow, focusing on the task instance restrictions for the restriction of roles, governance of access, and logs.

The authors in [33] discussed the IIoT vulnerabilities in the context of industrial processes. To make the business application more reliable, the authors proposed a framework based on blockchain that leverages machine learning algorithms to detect and mitigate attacks and security vulnerabilities in real-time. Blockchain technology was used for sensor access control management using smart contracts, and various machine learning algorithms such as ANN, SVM, DT, and naive Bayes were experimented with to validate the efficiency of the proposed framework. The authors in [34] also attempted to address the data breach vulnerabilities by proposing a deep learning privacy preservation framework. The framework safeguards the data by employing the attribute-based access control using the convolutional neural network (CNN). The proposed scheme considers the IIoT application for healthcare where massive data are produced and gathered. These data are used to explore the relationship between the users’ trust and their attributes using the CNN in this work. Similarly, to safeguard the data breach vulnerabilities and provide a better mechanism for data privacy in IIoT use cases and applications, the authors in [35] proposed a novel framework named ProModChain, which uses the Ethereum-based blockchain and federated learning to safeguard the privacy and trustworthiness of IIoT data. Federated machine learning is used to provide a global representation of the environment knowledge in distributed IIoT settings. The coordination between the private nodes is enforced using smart contracts for safety and transparency. Through the evaluation setup, the proposed model had significant results.

In [36], the authors leveraged machine learning for the role engineering process for access control. The authors argued that using access control as a frontline mechanism can ensure data privacy and integrity in critical infrastructures. However, in access control, the roles are manually extracted, which affects the efficiency and applicability of this approach. To reduce manual efforts, the authors employed Adaboost and SVM for the automatic role engineering process. Through evaluation experiments, the models presented good results. To further automate the access control mechanism, the authors of [37] leveraged a transformer-based deep learning approach to extract the access control policies from user and business stories. The authors argued that agile software development involves the user stories to incrementally develop the system, and the same idea can be employed to automate the policy specification. The proposed model takes inputs from the user stories and then detects if the provided input can be used for policy extraction or not. Further, it explores the actors, data objects, and their operational relationships to project them in the form of an access control policy.

The authors in [38] argued that critical data-intensive systems are always subjected to data access breaches while providing services to requests. To resolve these issues, the authors leveraged machine learning to propose a novel framework that is risk-adaptive. The proposed framework evaluates the genuineness of the requester and then calculates the risk attached to resolving the request. The proposed framework considers many contextual features of the requester in real-time such as the time, location, and previous history of the requester to calculate the risk.

## 4. System Model and Problem Formulation

### 4.1. Definition 1: Network Hierarchy and Structure

**Figure 6:**
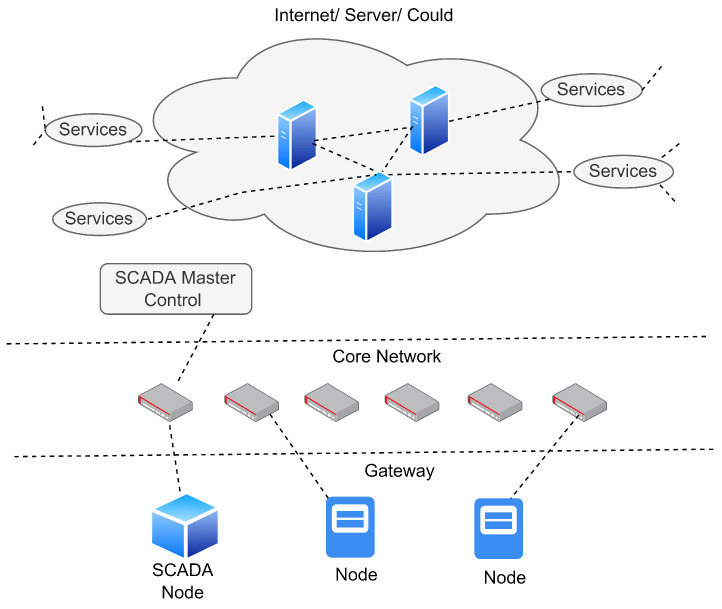
A representation of IIoT-based SCADA layered network environment.

In this work, we considered a network hierarchy in which each layer is connected to the others using a wireless access network (WAN), as shown in Figure 6. The top of the network consists of the main cloud, with multiple power stations, control units, management, and distribution services. Each service is responsible for performing tasks for SCADA applications such as monitoring and reporting the data back to the server to issue the alerts based on the network’s current state. The communication between the SCADA system and IoT devices is made possible by using the network nodes, which represent the specific region in the network. For each IoT device, communication would take place when the device successfully authenticates itself using its credentials. Afterward, the data are encrypted, and keys are assigned accordingly to help the IoT devices capture and transmit the data to the MTU control node.

### 4.2. Definition 2: Structure Mapping to Object States

The MTU contains a collection of services that are used to measure and deploy smart decisions on IoT devices. Such devices connected to the MTU using the WAN can be represented as a set N=1,2,3,…,n. In dynamic environment settings such as metro transportation systems, IoT devices that capture the data can contain a wide range of heterogeneous objects, which may be basic or intelligent depending upon the type of device. The main classes of these objects can be surveillance cameras, smoke and fire detectors, security checkpoints and emergency alarms, etc. For each device participating in the network, the classes of these objects can be represented as $U_{N}=1,2,3,\ldots ,u$.

### 4.3. Mapping States to Roles

The environment condition in which the IoT devices can reside will directly impact the state of the device as it will be capturing the data about it. For each device n, the state of the node at time instance *t* can be formally described as $S_{n^{T}}=\left\{x:x\in R\right\}$. These states of each device are further mapped to specific roles that define the permissions for the device to use the resources and services of the MTU in an adaptive and dynamic fashion. The following section describes the further operations in detail.

## 5. Proposed Role Engineering Approach

The approach to mapping the attribute-based access control is intuitive. At any time, for instance, t, the state of the SCADA node in the large-scale and fast-changing environment can be represented as the attributes of the nodes. The mapping of these node states to attributes can be formulated as: $A_{N}^{T}=A\leftarrow \;S:\;S,A\in R$ However, the attributes represented can be dynamic and static based on the type of information of the SCADA device. For example, if there are $A_{\left(m+n\right)}$ attributes for each, m is the static attribute such as the position of the device and n is the dynamic attribute such as the time of the day. The conventional RBAC approach lacks automation in role assignment. However, to obtain effective, yet efficient access control in a large-scale and dynamic environment, the integration of the advantages offered by both RBAC and ABAC can be promising. To build such a system, these attributes can be mapped to a finite set of roles using machine learning approaches. One issue that relates to ABAC is that automatic role propagation can lead to role explosion, where there are too many roles and required permissions in a large-scale enterprise. Nevertheless, this issue can be resolved in the integration of RBAC and ABAC and can be referred to as a hybrid access control mechanism. This proposed hybrid integration is efficient and effective in a dynamic environment. Based on the availability of the user, attributes, and roles, RBAC is applied to static attributes, and ABAC is applied to dynamic attributes. In that way, the hybrid model will be less computational complex. For example, for m+n attributes in the hybrid model, the result will be $2^{m}$ roles and $2^{n}$ rules, making it less complex than RBAC with $2^{\left(m+n\right)}$ roles and ABAC with $2^{\left(m+n\right)}$ rules, as follows:

(1) $$2^{m}+2^{n}<2^{\left(m+n\right)}:m>0,n>0$$

We can develop the hybrid context-aware access control with automated role engineering. Knowledge propagation and role assignment can be achieved by leveraging machine learning. For a machine learning model, the input is the combination of both static and dynamic attributes and the output is the decision of the model by inferring what role should be assigned to users’ attributes with a set of allowed permissions. The role of the machine learning model is to approximate a function that maps the attributes to role assignment with a set of permission. The weights for models can be learned initially by the manual role–attribute structure setups for IoT devices in the network.

### Machine Learning for Role Engineering

In this environment, different sensing nodes can capture different types of data corresponding to the environment application requirements. These captured data can be accessed by the set of users that have the access right privileges. Combining this with RBAC, the different users have different attributes that can be leveraged to determine the role of the user [39]. In the literature, this concept of mapping the dynamic characteristic of users to determine the role is referred to as the fine-grained access control (FGAC) model. In a WSN, the goal of FGAC is to map the unique privilege right to the user or end device based on attributes to access the piece of information [40].

Based on the availability of the manual user–role relationship by mathematical proof, the optimality of the automated role assignment can be guaranteed. However, the automated role assignment cannot guarantee the exposure of the system to various attacks such as denial of service, insider attack, and man in the middle. This problem can be resolved by using attribute-based encrypted systems to provide a safeguard against such attacks [41]. In such systems, integrating the machine-learning-based automated role assignment can provide accurate modeling of user–role relationships, making the system efficient and effective in terms of time and cost. In large-scale scenarios where roles are not manageable, fine-grained access policies better serve the purpose. The application scenario of this paper is to apply the role assignment for fine-grained access control based on encrypted data in mobile edge computing, but this scenario can be altered to encrypted sensory data of SCADA-based systems. The tailored scenario is similar in terms of SCADA sensors sharing the data with the edge server and a piece of that information is shared with the reporting authorities [17].

The key idea behind the automated role assignment is to learn the sensing data patterns and predict the end device node based on the characteristics the end device can have at any time instance *t*. For this purpose, different machine learning classifiers can be leveraged to learn the user–role relationship and predict the roles at runtime by analyzing the context of the end device attributes. In [36], the authors leveraged Adaboost and SVM to predict the device roles and automated role propagation. The authors discussed that, since the sensors’ data are usually not well separated, especially in the IoT environment, a predictive model can suffer from high variation in the results due to uncertainty lying in the data since they are not well separated. In this paper, we extended the work of [36] by leveraging the feedforward network (multi layer perceptron) and extreme learning machine (ELM) for this task along with conventional machine learning models.

## 6. Materials and Methods

### 6.1. Multilayer Perceptron

**Figure 7:**
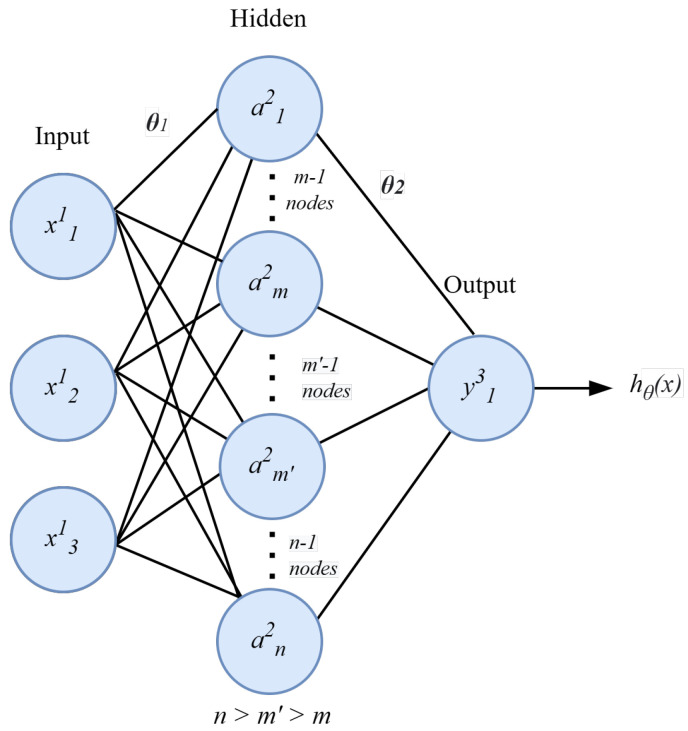
An abstract view of multilayer perceptron model architecture.

Multilayer perceptron (MLP) is a feedforward neural network that can be used for nonlinearly separable data. It uses three types of layers, i.e., input, hidden, and output layers. Figure 7 shows the architecture of the MLP model. Each layer in this model is responsible for processing the data and assigning the corresponding weights to it. The input layer has the input data attributes to send them with some assigned weights to the hidden layer for further processing after applying the activation function. The role of the activation function is to introduce the nonlinearity in the model fitting to make it able to capture the generic fitting on the data, as shown in Equation (3).

The initial weight calculation for the input layer is shown in Equation (2).

(2) $$net_{h}=w_{1}\left(x\right)+b_{1}$$

(3) $$out_{h}=\frac{1}{1+e^{-net_{h}}}$$

The weight calculation for the hidden layer is shown in Equation (4).

(4) $$net_{o}=w_{2}\left(out_{h}\right)+b_{2}$$

The output layer predicts or classifies the data as shown in Equation (5). The flow of the data is from the input to the output layer in the forward direction, like a feedforward neural network.

(5) $$out_{o}=\frac{1}{1+e^{-net_{0}}}$$

where *x* is the input and *w*_1_ is the weight for the first neuron in the model. Equations (2) and (4) calculate the hidden layer result and output layer result and then calculate the activation functions on these values. The required parameters for learning are $\theta =\{w_{1},w_{2},b_{1},b_{2}\}$. $w_{1}$ and $w_{2}$ are the weights to be learned. $b_{1}$ and $b_{2}$ are the bias, and Equations (3) and (5) are used to calculate the activation function. The most-used activation functions are the sigmoid used in the the equations and the tangent as tanh = $\frac{e^{a}-e^{-a}}{e^{a}+e^{-a}}$.

Equations (6) and (7) update the weight and bias.

(6) $$w_{1}\to w^{{}^{\prime }}=w_{1}-\eta \frac{\partial C}{\partial w_{1}}$$

In Equation (6), $w_{1}^{{}^{\prime }}$ is the updated weight, $w_{1}$ is the previous weight, and *C* is the total cost on an output neuron at the output layer.

(7) $$b_{1}\to b^{{}^{\prime }}=b_{1}-\eta \frac{\partial C}{\partial b_{1}}$$

Bias $b_{1}$ can be updated using Equation (7). The updated bias is $b_{1}^{{}^{\prime }}$, and $b_{1}$ is the previous bias, and *C* is the total cost. The error on the output layer can be calculated directly by using $C=C(out_{o})$. The proposed MLP algorithm is shown in Algorithm 1.
**Algorithm 1:** Multilayer perceptron.Forward pass(x,y,g(x))    Initialization of input data *x* and output vector *y*    g(x) is the chosen activation function    $\mathrm{Set}a_{i}\leftarrow \;x_{i}\mathrm{for}neuronsi=1,2,\ldots ,N$    $\mathrm{Set}b_{i}^{\prime }\leftarrow \;b_{i}\mathrm{for}neuronsi=1,2,\ldots ,N$    **Set** $net_{i}\leftarrow \;w_{i0}+\sum_{j\in pred_{\left(i\right)}}\;w_{ij}.a_{j}+b_{i}^{\prime }$ **for** all the hidden and output neurons *i* at layer *j*     $a_{i}\leftarrow g\left(net_{i}\right)$ **for** all the hidden and output neurons *i* at layer *j*    $\mathrm{Set}y_{i}\leftarrow \;a_{i}\mathrm{for}outputneuronsi=1,2,\ldots ,N$     **return** yBackward pass$\left(S,w^{\prime },\eta \right)$     Initialization of training samples *S*, updated weight vector $w^{\prime }$,     η is the chosen learning rate     $w_{k}\to w_{k}^{\prime }:=w_{k}-\eta \frac{\delta C}{\delta w_{k}}$ **for** each $k\in w_{i}$     Repeat until convergence     **return** $w^{\prime }$

### 6.2. Extreme Learning Machine

**Figure 8:**
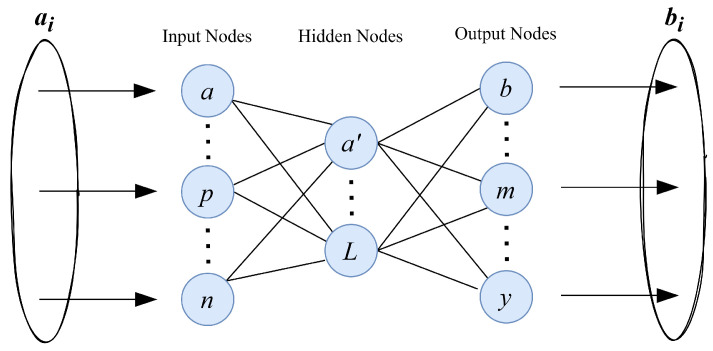
An abstract view of extreme learning machine architecture.

ELM, proposed by [42], is a single hidden layer feedforward neural network (SLFN) with at most *N* hidden nodes and with any nonlinear activation function, as shown in Figure 8. The key intuition behind this structure is that a single hidden layer having *N* nodes with a nonlinear activation function can exactly learn *N* unique observations of data in much less time. However, it was shown in [43,44] that using ELM with one single hidden layer with N nodes can exactly learn the unique data observations not only in a fast manner, but also providing generalized performance.

The ELM architecture has only three layers: The input layer, hidden layer, and output layer. In Equation (8), $i=\left[1,\ldots ,N\right]$ are the nodes in the hidden layer. *N* is the last node of the hidden layer. The architecture of ELM with a single hidden layer is shown in Figure 8.

The input weights and hidden layer biases can be chosen randomly if the activation functions of the hidden nodes are infinitely differentiable. The output weights can be analytically determined by simply calculating the generalized inverse (Moore–Penrose) of the hidden output matrices. For the weight calculations for *N* arbitrary unique data samples $\left(x_{i},t_{i}\right)$ that can be represented as $x_{i}={\left[x_{i1},x_{i2},x_{i3},\ldots ,x_{in}\right]}^{T}\in R^{n}$ and $t_{i}={\left[t_{i1},t_{i2},t_{i3},\ldots ,t_{in}\right]}^{T}\in R^{m}$, the standard single-layer feedforward net with *N* nodes and activation functions g(x) can mathematically be modeled as

(8) $$Z=\sum_{i=1}^{N}\beta_{i}.g\left(x_{j}\right)=\sum_{i=1}^{N}\beta_{i}.f_{i}\left(w_{i}.x_{j}+b_{i}\right)=h\left(x\right)\beta$$

where j=1,2,3,…,N and $w_{i}={\left[w_{i1},w_{i2},w_{i3},\ldots ,w_{in}\right]}^{T}$ is considered as a weight vector that will connect the input layer to the *i*th hidden node. Furthermore, $\beta_{i}={\left[\beta_{i1},\beta_{i2},\beta_{i3},\ldots ,\beta_{in}\right]}^{T}$ is the weight vector connecting the *i*th hidden node with the output layer’s nodes, and $b_{i}$ is the bias value for the hidden nodes.

The standard architecture of ELM with a single hidden layer with the activation function can approximate the *N* input sample with total zero error, meaning that $\sum_{i=1}^{N}\left\|h\left(x\right)\beta -t_{j}\right\|=0$ for $\exists \;\beta ,\;w_{i}\;and\;\;b_{i}$ such that

(9) $$\sum_{i=1}^{N}\beta_{i}.g_{i}\left(w_{i}.x_{j}+b_{i}\right)=t_{j}\;where\;j=1,2,3,\ldots ,N$$

By performing the mathematical substitutions, Equations (8) and (9), we can rewrite these equations in a simple form as below:

(10) $$H\beta =T\Leftrightarrow \beta =H^{+}.T$$

where

(11) $$H={\left[\begin{array}{ccc} g(w_{1}.x_{1}+b_{1}) & \cdots & g(w_{M}.x_{1}+b_{M})\\ \vdots & \cdots & \vdots \\ \vdots & \cdots & \vdots \\ g(w_{1}.x_{N}+b_{1}) & \cdots & g(w_{M}.x_{N}+b_{M}) \end{array}\right]}_{N*M}$$

and

(12) $$\beta ={\left[\begin{array}{c} \beta_{1}^{T}\\ \vdots \\ \vdots \\ \beta_{M}^{T} \end{array}\right]}_{M*m},\;\;\;T={\left[\begin{array}{c} t_{1}^{T}\\ \vdots \\ \vdots \\ t_{M}^{T} \end{array}\right]}_{N*m}$$

where *H* is called the hidden layer output matrix of the the network, and the *i*th column of the matrix *H* is the output of the *i*th hidden node of the network with respect to the input $x_{1},x_{2},x_{3},\ldots ,x_{N}$. In practice, to train the ELM model, the ultimate goal is to find the specific vectors $w_{i}^{{}^{\prime }},\;b_{i}^{{}^{\prime }},\;and\;\beta_{i}^{{}^{\prime }}\left(i=1,\ldots ,N^{{}^{\prime }}\right)$ such that

(13) $$\begin{array}{l} ∥\begin{array}{c} H\left(w_{1}^{{}^{\prime }},\ldots ,w_{N}^{{}^{\prime }},\;b_{1}^{{}^{\prime }},\ldots ,b_{N}^{{}^{\prime }}\right)\beta^{{}^{\prime }}-T \end{array}∥=\\ \;\min_{w_{i},b_{i},\beta_{i}}∥\begin{array}{c} H\left(w_{1}^{{}^{\prime }},\ldots ,w_{N}^{{}^{\prime }},\;b_{1}^{{}^{\prime }},\ldots ,b_{N}^{{}^{\prime }}\right)\beta^{{}^{\prime }}-T \end{array}∥ \end{array}$$

which is equivalent to the following cost function:

(14) $$E=\sum_{i=1}^{N}(\sum_{i=1}^{N^{\prime }}\beta_{i}g\left(w_{i}.x_{j}+b_{i}\right)-t_{j}{)}^{2}$$

### 6.3. Implementation

**Figure 9:**
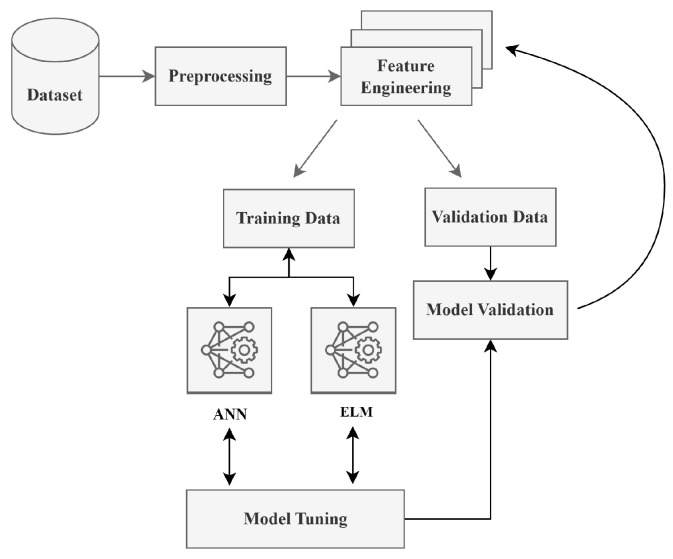
Machine learning implementation pipeline.

The core functionality of SCADA is to capture the data from the end devices and to provide the monitory control using host controller software. To leverage the data containing the static and dynamic attributes of the end devices, this paper leveraged the above-mentioned classifiers to learn and predict the roles. These learned roles will then be propagated to overcome the challenges and limitations posed by the manual role settings by the administrators. To make use of the MLP and ELM classifiers, this paper leveraged Algorithms 1 and 2, as shown in Figure 9.
**Algorithm 2:** Extreme learning machine.**Require:** Feature data of SCADA devices and their labels: $Z=\left(X_{m},y_{m}\right)\in R^{n},m\in \left[1,M\right],where\;X_{m}$ is a D-dimensional feature, consisting of sensing data, time, location, etc.**Ensure:** The final relevance vector for the $n_{th}$ device over the *K* pre-defined roles/permissions is $F_{m}=\sum_{i}^{N}g\left(w_{i}.x_{j}\right)$. The label of the final assigned roles is the largest value for $argmax_{r\in [1,2,\ldots ,N]}\left(F_{m}\right)$.**ELM**($X,t_{j},g\left(x\right),N^{\prime })$$Where\;X=\left(x_{i},t_{j}\right)|x_{i}\in R^{n},\;t_{j}\in R^{m},\;i=1,2,\ldots ,N$*y* is the output vector,g(x) is the chosen activation function, and $N^{\prime }$ is the number of hidden nodes.Set $a_{i}\leftarrow \;x_{i}$ **for** input neurons i=1,2,…,NSet $b_{i}^{\prime }\leftarrow \;b_{i}$ **for** input neurons i=1,2,…,NSet $h_{i}^{\prime }\leftarrow \;w_{i0}+\sum_{j\in pred_{\left(i\right)}}\;w_{ij}.a_{j}+b_{i}^{\prime }$ **for** all the hidden and output neurons *i* at layer *j*$h_{i}\leftarrow g\left(h_{i}^{\prime }\right)$ **for** all the hidden and output neurons *i* at layer *j*$\beta_{i}\to \beta_{i}^{\prime }$ **for** all output neurons i=1,2,…,N$T_{i}\to t_{i}$ **for** all output neurons i=1,2,…,NCalculate $\beta =H^{-1}.T$Calculate the label output of classifier $y^{\prime }\leftarrow argmax_{r\in [1,2,\ldots ,N]}\left(F_{m}\right)$**return** $y^{{}^{\prime }}$

#### SCADA Dataset

In order to validate and test the proposed methodologies, a benchmark dataset was required for the experiments. Though many benchmark datasets are available for public use, a more concrete dataset by [36] was used in this paper to conduct the experiments. The dataset created by [45] contains more dynamic features of SCADA end device signatures, and more randomness is introduced in the dataset, which is beneficial from the perspective of more generalized deep learning model fittings. This is important because the order in which the data observations are fed to the models affects the decisions, especially in neural networks. Further, the dataset does not have any correlation between the labels and features in it as compared to the previous existing datasets. With correlated data, the supervised model shows poor performance regardless of how deep the model is. In that case, linear regression is well suited. A thorough comparison of different datasets is available in the study [45], which justifies the need to use these data for experimental purposes.

The benchmark dataset contains 27,464 records of the device transactions on the network. Each record consist of a 17-dimensional feature space containing the network transaction value, including the network address, function, and command payload along with the labels. A complete feature list is presented in Table 1. In a system where role propagation is required to be automated, these features can be replaced with the fine-grained attributes of the devices such as time, location, topological structure, type, etc., and the labels can be the desired roles. The dataset is publicly available, and the creation process was discussed by the author in the paper [45]. However, this dataset has been used by many studies for many applications [46,47].

## 7. Results and Discussion

To test and validate the hypothesis, we used all the features of the dataset mentioned in Table 1. Data wrangling was performed on the features to replace the missing values of features to achieve better classification accuracy. The missing values were replaced using the imputing method by keeping the prior and existing values for each feature. To make each class observation well separated from the other, feature scaling was performed due to the single-variate time series nature of the data. To perform the feature scaling, a standardization approach was used where each feature was subtracted from its mean value and divided by the standard deviation. The transformed tidy data were then used for training and testing the hypothesis of this paper.

### 7.1. Hyperparameters

There are 274,628 class observations in the dataset, and each class representation is shown in Table 2 in detail. For the training of the model, the category labels were used, which consisted of eight different classes, as shown in Table 2. To carry out the experiments, data splitting was performed as 60%, 20%, and 20% for the training, validation, and testing of the model. Further, the MLP model was implemented using a five-layered feedforward architecture with ReLU as the activation function at the input and hidden layers. A detailed summary of the model architecture is shown in Table 3.

The model was trained using 55,035 trainable parameters for 50 epochs with the Adam optimizer using a $1\times 10^{-3}$ learning rate with no decaying average of the gradients. The obvious reason behind using the Adam optimizer is that it uses the weighted average of the gradients, which tends to converge faster than the traditional gradient descent approach. For each epoch, a batch size of 32 was used for the training and validation of the model fitting on the data to make the process less computationally expensive. To calculate the validation and training loss in each epoch, the categorical cross-entropy was used as the loss function. The obvious reason behind choosing a loss function as the categorical cross-entropy is the multiclass one-hot encoded target vectors.

### 7.2. Model Training and Validation

**Figure 10:**
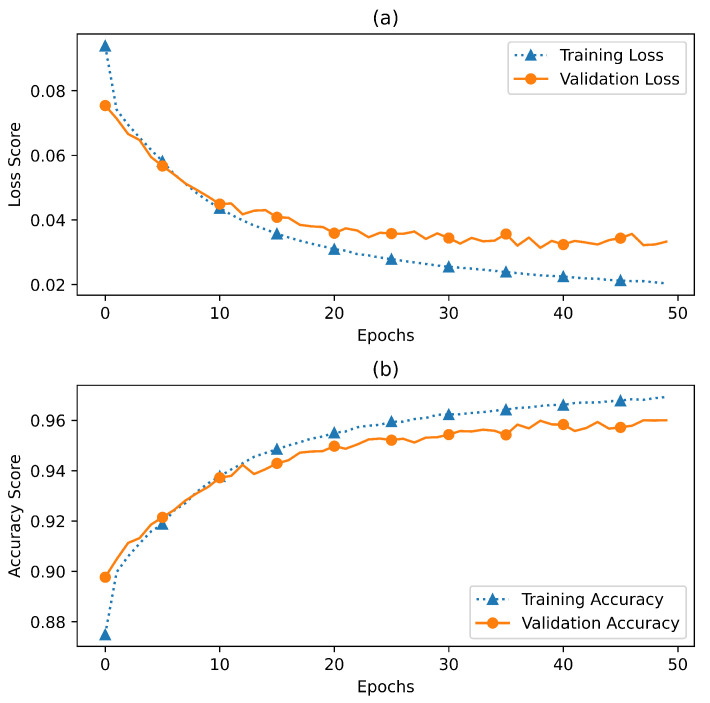
MLP learning and validation curves.

The model fitting on the training and validation set is shown in Figure 10. The selection of the hyperparameters was based on a hit and trial strategy, where the model was rigorously trained and evaluated using different epochs. The maximum training accuracy of 97% was achieved for 50 epochs, and the validation accuracy was at 96%. Figure 10 gives a detailed insight into the validation accuracy with respect to the training accuracy. After training the model, the test data were used for the evaluation of the model. The model performed well and achieved a test accuracy of 96% with a 93% precision, 88% recall, and 91% F1-score. Table 4 shows the detailed intra-class precision, recall, and F1-scores for the testing data.

**Figure 11:**
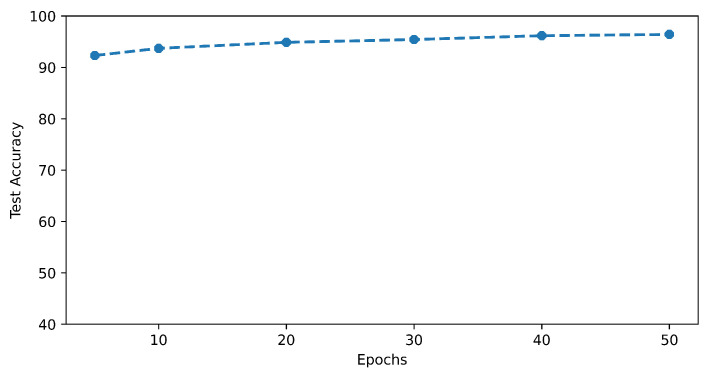
MLP test accuracy relationship with training for different numbers of epochs.

Similarly, we evaluated the test accuracy trend of the model using the different numbers of epochs for training while keeping the batch size of 32 for each number of epochs. Figure 11 shows the plot of the test accuracy behavior of the model when it was trained for a different number of epochs. To carry out this experiment, the model weights needed to be dealt with with care as previously fine-tuned weights can make the training of the next iteration biased. To avoid this phenomenon, the model’s initial weights were preserved after compilation and reused in each iteration of training using different epoch numbers.

The model was trained for E=10,20,30,40,50, where *E* represents the epoch number for each training iteration. The test accuracy against each *E* is shown in Figure 11. Further, ELM was also leveraged using the same data sampling strategy. The main difference of ELM with a single-layer feedforward network is the usage of the random weights and threshold values for hidden nodes between the input and hidden layer rather than using gradient functions for local maxima. The output weights are then derived by solving the linear matrix equations. Due to this core difference, ELM tends to converge much faster than backpropagation-based networks with a high degree of generalization and with an acceptable extent of accuracy as well. However, the performance of the ELM classifier is heavily dependent on the hyperparameters such as the hidden number of nodes, the activation functions to induce nonlinearity in the feature maps between the layers, and the randomization range of the threshold values of the hidden nodes. Similarly, the randomization range of the weights between the input and hidden layer is also crucial for the classifier to achieve the desired accuracy results. With different ranges of values being used for hyperparameter tuning, ELM tends to show high variance in the results.

In the study [48], the authors tested the ELM classifier using the randomization range for the weights between the input and hidden layers as $\left[-1,1\right]$ and $\left[0,1\right]$ as the threshold range value for the hidden nodes. We tested the claims of this study in this paper using the sigmoid and ReLU activation functions. The performance of ELM was slightly better using the sigmoid than ReLU, which authenticates the claims of the study. One major reason behind this phenomenon can be that ELM is sensitive to the data distribution and activation function transformations. Each activation function feature mapping would eventually be different, making ELM show high variance results.

### 7.3. Comparative Analysis

**Figure 12:**
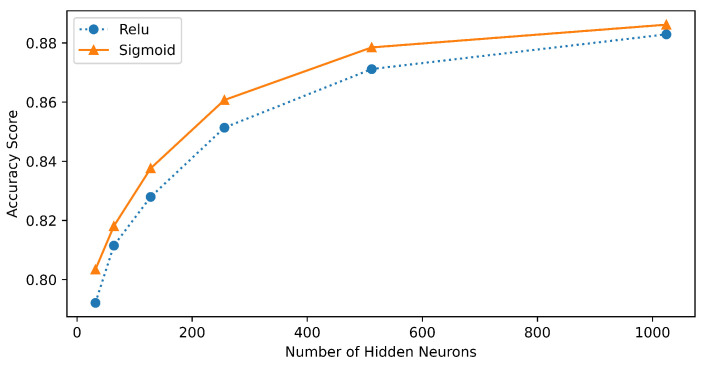
Performance comparison of ELM with different numbers of hidden nodes and activation functions.

Selecting an appropriate number of hidden nodes is also crucial to achieve a good convergence of the model on the data. To visualize this phenomenon, we tested the performance of the ELM classifier using both the sigmoid and ReLU activation functions with a varying number of hidden nodes. Figure 12 shows the relationship between the test accuracy scores with the number of hidden nodes. From Figure 12, it can be inferred that the accuracy increased with an increasing number of hidden nodes in the model architecture. With that, in a careful manner, it can be said that there exists a trade-off between the computationally expense of the model and the accuracy.

**Figure 13:**
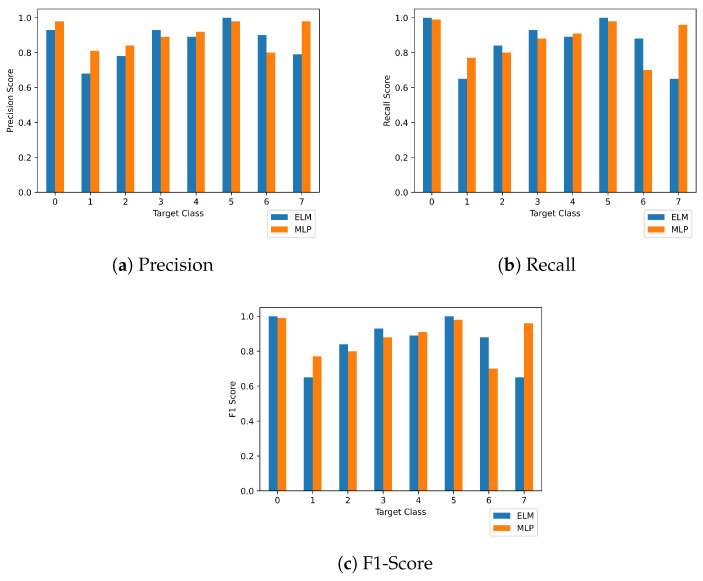
Comparison of accuracy metrics for MLP and ELM for each target class in the dataset.

The right balance between both variables can be defined as an acceptable score of accuracy with available computational resources. Further, for the given test data, ELM showed an overall accuracy of 89%, with 88% precision, 85% recall, and 86% F1-score, respectively, using 1024 hidden nodes. To obtain these results in our experiments, we used the singular-value decomposition method to find the pseudo-inverse matrix of the weights due to it being more computationally efficient than the least-squares method. The intra-class scores of precision, recall, and F1 are represented in Table 5 for the ELM predictions on the test data. From Table 4 and Table 5, it can be inferred that MLP outperforms ELM in better classification convergence for the SCADA dataset, but ELM shows better time efficiency than MLP in terms of model throughput for the training and testing process. The detailed comparison of the scores of both models is presented in Figure 13. As per our findings, the performance of ELM on highly sparse data for classification use cases is still uncertain and an open problem. There are certain frameworks presented in [49,50,51], but the cumulative agreement of the community is still uncertain. The time for training and testing for both ELM and MLP is presented in Table 6. As the architecture and hyperparameters for both models are different, non-overlapping, and fine-tuned for better results, the comparison presented in Table 6 can be used to compare the obtained results with the time consumption. Due to the stochastic nature of the weights’ initialization, different hyperparameters and architecture varieties can lead to wholly different performances. Table 7 represents the comparison of the proposed scheme with related work, and it can be inferred from the table that the proposed pipeline outperformed the related work in terms of accuracy. One reason behind this is that deep models tend to show better mapping and modeling of large data. The representation of features is crucial for better fitting of the curve to classify. However, the ANN was better in terms of accuracy, but its computational overhead was also greater than the other models. A trade-off can be decided between the accuracy and time overhead depending on the business use case.

## 8. Conclusions and Future Work

The objective of this study was to leverage machine learning models for automated role propagation for fine-grained access control in SCADA-based IIoT use cases. However, due to the nonavailability of the benchmark dataset for access control, a benchmark dataset of the SCADA system was tailored in this study to test and validate the hypothesis of this study. We proposed a practical approach of using machine learning for automated role engineering with encrypted sensory data in SCADA-based applications. In our experiments, MLP outperformed the ELM model with more accurate results, but the convergence efficiency with respect to time was better in the ELM’s application. For future work, we would consider the following directions:

1. There is still an open problem of collecting data samples from SCADA system applications with feature, role, or permission tuples in real-life use cases such as smart transportation, smart healthcare, etc.
2. Customized data can be further investigated using advanced tailored machine learning algorithms such as multilayer extreme learning machines and hybrid deep models to achieve robust accuracy for role engineering and propagation in fine-grained access control.
3. Similarly, based on the availability of metadata and the structural topology of sensory devices, recent language models can also be employed to derive robust role engineering in this domain.
4. Likewise, the role of policies in the access control model is also very crucial in this domain. As for future work, we will explore the application of machine learning to effectively map the non-conflicting policies to manage the attribute-based access control mechanism for IIoT use cases.

## Figures and Tables

**Table 1:** A complete feature list of the network payload in the dataset.

Sr.	Feature	Type	Sr.	Feature	Type
1	address	network	11	control scheme	command payload
2	function	command payload	12	pump	command payload
3	length	network	13	solenoid	command payload
4	setpoint	command payload	14	pressure	response payload
5	gain	command payload	15	crc	network
6	reset rate	command payload	16	command rate	network
7	deadband	command payload	17	time	network
8	cycle time	command payload	18	binary	label
9	rate	command payload	19	categorized	label
10	system mode	command payload	20	specific mode	label

**Table 2:** Types and categories of attacks present in the dataset.

Sr.	Attack Type	Acronym/Label	#	Category
1	Normal	Normal(0)	1333	Normal Payload
2	Naive Malicious Response Injection	NMRI(1)	7753	Response Injection
3	Complex Malicious Response Injection	CMRI(2)	13,035	Response Injection
4	Malicious State Command Injection	MSCI(3)	7900	Command Injection
5	Malicious Parameter Command Injection	MPCI(4)	20,412	Command Injection
6	Malicious Function Code Injection	MFCI(5)	4898	Command Injection
7	Denial of Service	DoS(6)	2176	Denial of Service
8	Reconnaissance	Recon(7)	3874	Reconnaissance

**Table 3:** Detailed model summary of multilayer perceptron network.

Sr.	Layer	Type	Output Shape	Parameters	Activation
1	Input	Dense	(None, 256)	(12,032)	ReLU
2	Hidden	Dense	(None, 128)	(32,896)	ReLU
3	Hidden	Dense	(None, 64)	(8256)	ReLU
4	Hidden	Dense	(None, 32)	(2080)	ReLU
5	Output	Dense	(None, 8)	(264)	Softmax

**Table 4:** Intra-class summary of MLP performance on testing data.

Sr.	Label	Precision	Recall	F1
1	0	97%	99%	98%
2	1	86%	77%	81%
3	2	88%	80%	84%
4	3	91%	88%	89%
5	4	94%	91%	92%
6	5	99%	98%	98%
7	6	92%	70%	80%
8	7	99%	96%	98%
Total		93%	88%	91%

**Table 5:** Intra-class summary of ELM performance on testing data.

Sr.	Label	Precision	Recall	F1
1	0	88%	100%	93%
2	1	72%	65%	68%
3	2	72%	84%	78%
4	3	92%	93%	92%
5	4	89%	89%	89%
6	5	100%	100%	100%
7	6	91%	88%	90%
8	7	100%	65%	79%
Total		88%	85%	86%

**Table 6:** Comparison of the time performance of both models on SCADA dataset.

Model	Time (s)	
	Training	Testing
MLP	812	5
ELM	57	1

**Table 7:** Comparison of the proposed model with related work [36].

Sr	Zhou et al. [36]		Proposed	
	Adaboost	SVM	ANN	ELM
Accuracy	79%	80%	96%	89%

## Data Availability

The dataset can be made available through the declaration of all the authors.
